# Patient-centered infertility questionnaire for female clients (PCIQ-F): part I: questionnaire development

**DOI:** 10.1186/s12874-021-01376-w

**Published:** 2021-09-20

**Authors:** Hana Hasan Webair, Tengku Alina Tengku Ismail, Shaiful Bahari Ismail, Azza Jameel Khaffaji, Nik Hazlina Nik Hussain, Azidah Abdul Kadir, Rosediani Muhamad, Fatin Aina Abu Bakar, Nur Raihan Ismail, Nagwa Badri

**Affiliations:** 1 Department of Family Medicine, School of Medical Sciences, Universiti Sains Malaysia, Health Campus, 16150 Kubang Kerian, Kelantan Malaysia; 2 Department of Family Medicine, Hadhramout University, College of Medicine, PO Box 50512, Mukalla, Hadhramaut Yemen; 3 Department of Community Medicine, School of Medical Sciences, Universiti Sains Malaysia, Health Campus, 16150 Kubang Kerian, Kelantan Malaysia; 4 Obstetrics and Gynaecology Department, Ministry of Health, King Abdulaziz Hospital, P.O.Box 31467, Jeddah, 21497 Saudi Arabia; 5 Women’s Health Development Unit, School of Medical Sciences, Universiti Sains Malaysia, Health Campus, 16150 Kubang Kerian, Kelantan Malaysia; 6 Johor Bahru District Health Office, Johor Bahru, Malaysia; 7 International Medical Center, Jeddah, Saudi Arabia

**Keywords:** Infertility, Patient-centered, Questionnaire

## Abstract

**Background:**

Patient-centered care is an essential component of health care quality. To achieve patient-centered care, health care authorities should have a clear definition and an applicable tool to measure the extent of its application. The real concept of patient centeredness should be developed by the patients themselves. We aimed to demonstrate a way to develop a draft Arabic patient-centered infertility care (PCIC) questionnaire for females clients following practical steps that address women with infertility.

**Methods:**

An iterative process of questionnaire development was undertaken by combining two approaches: the steps proposed by Robert F. DeVellis for scale development and the recommended practices for questionnaire development and testing in the European statistical system. We attempted to develop the draft questionnaire that involved conceptualization and operationalization, generation of an item pool, development of the questionnaire format, review of the initial item pool by experts, and consideration of validation items for inclusion.

**Results:**

We generated an item pool from in-depth interviews with 14 women who sought infertility care within 6 months before the interview time. We then added more items from a literature review. The item pool contained 123 items distributed through 10 domains. Ten women with infertility were included for face validation. Then, experts with backgrounds in Obstetrics and Gynecology, Family Medicine, and Public Health reviewed the item pool using content validation (*n* = 10 professors and/or specialists). The item pool was finally reduced to 57 items. We developed the draft Arabic patient-centered infertility care questionnaire for female clients (PCIQ-F) with three sections, including 66 items: background variables, PCIC experience variables, and a general question about the quality of infertility care in the health facility. The draft questionnaire was further reviewed and edited last by experts in preparation for part 2, which will test the questionnaire and prepare the final version.

**Conclusion:**

The PCIQ-F questionnaire development is a multi-step iterative process started and ended by the target users as experts. Experts’ participation in infertility care and in questionnaire format development had a great impact on questionnaire development and conflict resolution. We recommend this transparent and replicable approach for new instrument developers; it is likely to generate a questionnaire that is valid and acceptable to target users. The draft PCIQ-F questionnaire is ready for testing of its psychometric properties before the final version to measure the PCIC level in health facilities.

**Supplementary Information:**

The online version contains supplementary material available at 10.1186/s12874-021-01376-w.

## Background

Patient-centered care (PCC) is the sixth element of health care quality, as reported by the National Academy of Medicine (formerly called the Institute of Medicine) [1]. To ensure that PCC is actually implemented in health care settings, new measures should be developed, tested, and piloted [2]. These measures should include actionable feedback from relevant stakeholders in the development stage to achieve patient centeredness [2]. Infertility, which is defined as the inability to conceive after 12 months of unprotected intercourse, is a public health concern associated with psychological distress, economic burden, and poor quality of life [3–5]. Infertility has affected approximately 19% of couples in Saudi Arabia [6]. Infertility care is known for its long-lasting and heavy physical and emotional burdens on the affected couple, making PCC of paramount importance [7, 8].

A systematic review, including 51 studies, by Dancet et al. studied patients’ perspectives on fertility care [9]. They found significant methodological limitations, and the majority of the reviewed studies did not examine patient perspectives as their primary objective. An important limitation was the lack of questionnaire validation in the majority of studies. After Dancet’s review, new assessment tools were developed and validated in relation to infertility care. However, many of these tools were focused on other aspects related to infertility care but not PCC, such as the Fertility Quality of Life tool [8] and Cardiff Fertility Knowledge scale [10]. The patient centeredness questionnaire-infertility (PCQ-infertility) by van Empel et al. in 2010 [11] is one of the most widely used questionnaires to objectively score the level of patient-centered infertility care (PCIC). The drawback of this tool is that it is directed at infertile couples together, even though it is known that each partner has their own concerns and needs during their fertility care journey. Another concerning point is that the tool is being developed and validated with European patients, so it might not be applicable to a different culture. Holter et al. developed and validated a questionnaire for both women and men, but it was specific to in vitro fertilization (IVF) treatments. Additionally, it was based on the theoretical foundation of the general instrument, which was quality from a patient’s perspective but not from the infertile patients’ perspectives [12]. Our literature review justified the need of a new tool for measuring PCIC from Arab clients’ perspectives. The dynamic nature of content validity due to the emerging data and theories over time also suggested the need for periodic revision of psychological assessment instruments to ensure that they can measure the targeted construct [13].

The study aim was to develop a new tool to assess PCIC for female clients (PCIQ-F) based on the experiences of Arab women.

## Methods

**Fig. 1:**
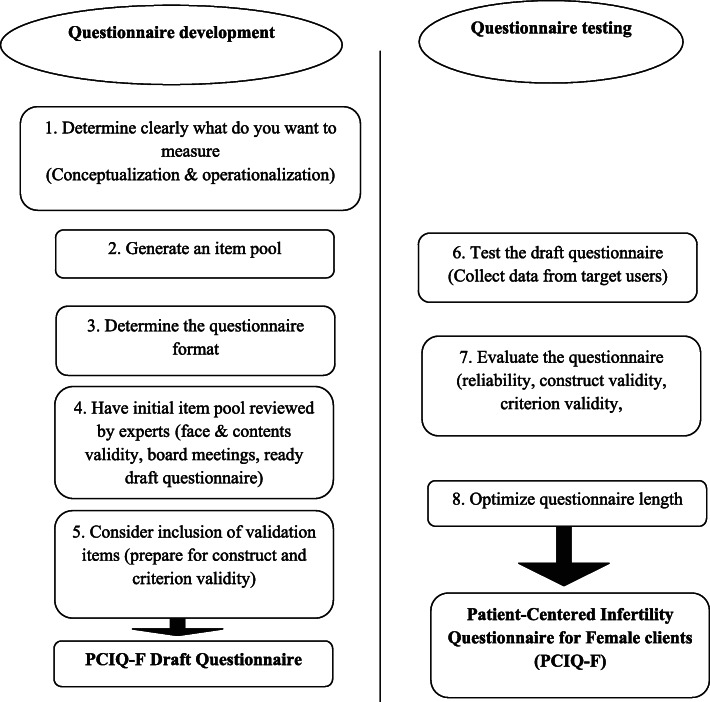
Questionnaire development and testing steps

We developed the PCIQ-F following the steps proposed by Robert F. DeVellis for scale development [14] and the recommended practices for questionnaire development and testing in the European statistical system [15]. The steps of questionnaire development (part I) and questionnaire testing (part II) are summarized in Fig. 1. This manuscript will cover questionnaire development (part I).

### Step 1: determine clearly what you want to measure

#### Specification of the survey objectives

We developed the questionnaire to objectively measure the level of patient centeredness of women’s infertility care provided by health facilities in Arabic countries.

#### Literature review

We started by reviewing the literature looking for available tools to achieve the questionnaire objectives. The review revealed many studies and systematic reviews conducted on the topic in Western countries [11]. To the best of our knowledge, no articles measured or even defined PCIC from Arab patients’ perspectives comprehensively [16]. Patient centeredness is a culture-sensitive subject. Values of infertile Western couples may not be applicable in Arab populations, considering religious and cultural differences.

#### Conceptualization and operationalization

We adopted the concept of PCC of the Institute of Medicine “*Providing care that is respectful of, and responsive to, individual patient preferences, needs and values, and ensuring that patient values guide all clinical decisions*” [17]. In addition, Picker’s Eight Principles of PCC concluded that the concept has eight dimensions [18]. To apply the concept, we defined what Arab infertile women need, prefer, and value concerning infertility care. Their perspective represented the items and dimensions of the PCIQ-F. To operationalize the concept, we translated the items into questions asked about women’s recent experiences with infertility care focusing on the points mentioned as valuable and important to them. We grouped PCIQ-F items into dimensions, which represented the domains of the final questionnaire. Each question has a score, so the total scores will reflect the level of PCIC in general and in each dimension. We added a few questions about demographic and fertility care characteristics as a possible source of error [11]. This method was used by van Empel et al. to measure PCIC among European couples [11]. The developed questionnaire had been tested among different Western infertile couples and found to be a valid tool to assess PCIC [19, 20].

#### Exploring concepts

Due to the lack of a comprehensive definition of PCIC from Arab women perspectives [16], the concept of PCIC was explored through in-depth interviews (IDIs). The sample included 14 infertile Arab women who sought infertility care in Jeddah, Saudi Arabia within 6 months preceding the interview time. They were asked about their experience with infertility care and what they need, expect, prefer, and value. We chose IDIs to explore the concept because in the case of new survey development, it is recommended that a user-focused consultation be conducted to define the concept comprehensively [15]. The details of this qualitative study have been published in a separate paper [21].

### Step 2: generate an item pool

An item pool was generated by combining both inductive and deductive methods; i.e., from IDIs and a literature review. To capture the concept comprehensively, we developed a large item pool that included items revealing PCIC in different ways. Such apparent redundancy was helpful in this stage [14].

### Step 3: determine the format for measurement

#### Decision on the data collection mode

This was a self-administered Arabic questionnaire. The topic of measuring the degree of PCIC from the patient’s point of view requires some degree of confidentiality. The respondents should feel free to disclose their opinion without being influenced by the presence of medical staff or a third party [22]. The questionnaire generally asks about the patient’s experience and opinion about fertility care she received, so it asks about easily retrievable information. In addition, the questionnaire assesses the PCIC during a specified frame time (during the last 6 months of treatment) in a defined health facility to make it easier to memorize and give more accurate answers as the quality of health care is not a stable variable [14].

#### Writing the questions

Our questionnaire translated the variables into simple, understandable, and unequivocal questions in words taken from target users’ transcripts. Then, the questionnaire was checked for appropriateness by another group of target users in step 4 of questionnaire development (face validation). We invited target users in each and every step of questionnaire development to improve its validity and make it user friendly. Clear and short general instructions were written in the front page of the questionnaire to define some terms and guide the respondents. To avoid being overlooked, special instructions were written close to the question to which they refer.

Regarding the type of questions, apart from background questions, all the others are closed-ended ones. We did not use the simple agree/disagree Likert scale for all items but instead used an item-specific rating scale because that scale is more reliable [23] and helps avoid acquiescence-response bias (people like to be seen as agreeable) [23]. We tried our best to make the question stem as short and simple as possible. Regarding the number of response options, we used few options for each stem; i.e., 4–5 to avoid respondent fatigue especially because our questionnaire is long, which will help improve the discrimination ability of the respondents even with few response options [14]. Response options are ordered in an obvious continuum; e.g., from the lowest to highest to improve the respondents’ understanding.

We wrote questions in both positive and negative directions to minimize the possibility of set responses [14, 23] without using confusing negative phrasing. We did not use filter questions, which direct the respondent to different portions of the questionnaire according to their answer because they are often confusing [24].

#### Determining question sequences

Questions were arranged into logical domains by PCIC dimensions. The domains were ordered naturally following a logical stream similar to what patients will go through during their fertility care journey.

### Step 4: have initial item pool reviewed by experts

At this stage we involved two kinds of experts: lay and subject matter experts. We started by inviting lay experts to participate in face validation of the initial item pool. Those are women with fertility problems who sought medical care within 6 months prior to invitation time. Subsequently, we modified the initial item pool on the basis of the face validation results as discussed in the questionnaire development board, which was followed by content validation that included subject matter experts. Another meeting among research team members was held to discuss the input from content validation and develop the first draft of the questionnaire for step 6. The details of face and content validation follow.

#### Face-validation

Face-validity was assessed according to the Lynn criteria of content validity for instrument development [25]. We invited lay experts to participate in evaluating the initial item pool concerning importance and clarity. Lay experts were the same target users; i.e., infertile women who had experienced infertility care within the last 6 months before the date of invitation. We contacted the women who agreed to participate by phone and explained to them the purpose and process of face validation, and experts’ inclusion criteria. We motivated them by assigning them as experts who would participate in developing a tool that will be used to measure an important indicator of health care quality. As all the potential experts were Muslims, we added religious motivation words based on a core principle of Islam about helping other human being to get rewards from Allah. Adding to that, all lay experts received a letter of thanks for their time and efforts. Lay experts were recruited through purposive and snowball sampling methods. We invited 14 women by phone, and 11 of them agreed to participate. The invitations were extended starting in August until October 2019. The number of participants who agreed to participate was within the acceptable range of the FVI calculation [25]. We included a heterogeneous group of experts with diverse personal and infertility care characteristics to improve validity. We prepared an Arabic online questionnaire with three main sections: an introductory section, a personal information section, and an item-evaluation section. Section one included a brief description of the questionnaire, response instructions, and a consent statement. Section two included questions about date of birth, level of education, parity, number of living children, duration of infertility treatment, place of infertility care, type of infertility treatment used, and outcomes of treatment. Section three included the PCIC items; i.e., the generated item pool divided into domains. For each item in section three, there were two questions and a feedback instruction: “How do you rate the importance of the item to measure PCIC?,” “How do you rate the clarity and language of the item?,” and “Write your suggestions concerning this item.” For the questions on importance and clarity, we adopted a 4-point ordinal rating scale [9, 25]: 1) The item is not important (clear), 2) The item is somewhat important (clear), 3) The item is important (clear), 4) The item is very important (clear). We adopted a self-administered online method because of its preference for use with sensitive topics so that the respondents can disclose their opinions freely. In addition, it reduces the time and cost of data collection, permits reaching a wide range of respondents, and is associated with a high item-response rate [22, 26]. We applied “Forced answer” technology to avoid missing questions and maximize the item-response rate [27]. The online questionnaire was pre-tested for usability and for ensuring the least respondent burden until the final format was agreed upon. A maximum of 25 min was needed to complete the three parts, which was within the acceptable range [15]. The Google Forms platform was used to design the questionnaire in a manner that ensured respondent anonymity. A questionnaire link was sent to the respondents. As we did not include any identifying data of the participants, we received the forms on the spreadsheet anonymously. As the questionnaire was long, we sent it to the experts divided into three links to prevent participants’ fatigue. We sent a reminder after 2 weeks to maximize the response rate. All questions in section 3 (PCIC items) were labeled as compulsory so that the participant would not submit the form with missing questions.

For data analysis, we computed the FVI for each item (I-FVI) and the scale face validity index (S-FVI/Ave) [25, 28]. The I-FVI equals the number of participants who rated the item positively (3 or 4) divided by the total number of participants. The S-FVI/Ave was calculated as the sum of the I-FVI for all items divided by the number of items [29]. We adopted the S-FVI/Ave rather than the universal agreement method of the scale validity index, which equals the number of items that were rated positive (3 or 4) by all experts divided by the total number of items. For the scale content validity calculation, the scale content validity index (S-CVI/Ave) method is preferred over the universal agreement when the number of experts is high because reaching universal agreement is difficult [29]. We interpreted the findings on the basis of the Lynn criteria for the I-FVI; as a result, an excellent FVI was defined as an I-FVI of ≥0.78 for 10 experts [25] and an S-FVI/Ave of ≥0.90 [29]. For new instruments, Davis suggested ≥80% agreement among the judges [28]. After calculating the results, we held a meeting involving the questionnaire’s developers to discuss the FVI and asked them to suggest revisions, deletions, or substitutions.

#### Content validation

Ten experts in research methodology or infertility care were purposefully invited to participate in assessing content validity. The experts included two professors; one associate professor; three assistant professors; and four physician specialists in Family Medicine, Public Health, Obstetrics and Gynecology, and infertility who had experience in questionnaire development. The retained items after face validation were sent to the experts. Calculation and interpretation of the CVI for the items (I-CVI) and the scale (S-CVI/Ave) were conducted in the same manner as that used for the FVI [25, 28, 29].

### Step 5: consideration of the validation items for inclusion

For construct validity, the questionnaire comprised a domain (latent variable), items (observed variables), and participants’ characteristics (source of error).

For criterion validity, a general question about the quality of infertility care in the health facility was added to the questionnaire to measure concurrent criterion validity. PCC is known to be associated with higher health care quality [1].

### Ethics approval and consent to participate

All methods were performed in accordance with the Declaration of Helsinki. Ethical approval for the study was provided by the Medical Research and Studies Department, Directorate of Health Affairs, Ministry of Health, Jeddah, Saudi Arabia (number A00306), and the Universiti of Sains Malaysia (number USM/JEPeM/15020056). Written informed consent was obtained from all participants.

## Results

### Item pool

IDIs generated nine dimensions with a total of 116 items. We added seven more items from the literature review [8–12, 16]. The item pool contained 123 items and 10 domains (Additional file 1).

### Face-validity

The total number of returned questionnaires was 11. One was excluded because the respondent filled out only one part of the questionnaire because of an emerging social issue. The experts’ characteristics are presented in Table 1. Finally, 10 completed questionnaires were included in the analysis.

**Table 1:** Participants characteristics for face validation

Participant characteristics	Category	No.(%)
Age (years)	25-	3 (30)
	30-	3 (30)
	35-	2 (20)
	40-	1 (10)
	> 45	1 (10)
Education	Up to secondary school	3 (30)
	Bachelor	6 (60)
	Higher education	1 (10)
Type of infertility	Primary	4 (40)
	Secondary	6 (60)
Duration of infertility (years)	1-	5 (50)
	5-	3 (30)
	10–15	2 (20)
Duration of seeking care (years)	2	2 (20)
	4	3 (30)
	5	1 (10)
	6	2 (20)
	7	1 (10)
Treatment (total > 100% as several treatment modalities used)	IVF/ICSI^a^	5 (50)
	Ovulation induction	6 (60)
	Surgery	1 (10)
	IUI^b^	1 (10)
Outcome	Failed	7 (70)
	Miscarriage	2 (20)
	Pregnant	1 (10)
Health facility visited (total > 100% as more than one facility visited)	Polyclinics	3 (30)
	General government hospitals	3 (30)
	General Private hospitals	5 (50)
	Hospitals with ART^c^ Units	5 (50)
Total		10 (100)

^a^*IVF/ICSI* in vitro fertilization/intracytoplasmic sperm injection, ^b^IUI; Intrauterine Insemination, ^c^*ART* Assisted reproductive technology

Meetings among research team members were held to discuss these results, take action, and prepare the item pool for content validation. Based on our preset criteria, an I-FVI > 0.78 and an S-FVI > 0.90 were considered to be excellent. Results analysis showed that the I-FVIs of 123 items ranged from 0.3–1 and 0.4–1 for clarity and importance, respectively. Generally, 28 items were scored < 0.78 for clarity and/or importance. For clarity, 24 items scored < 0.78 and 7 items scored < 0.78 for importance. The S-FVI/Ave was a bit low for clarity (0.80) and excellent for importance (0.90) (Additional 1).

For items with an I-FVI < 0.78, the board members discussed whether to delete, or re-write according to the clarity, importance, notes from lay experts, and panel members suggestions. All items with a low importance score were deleted. Items with a low clarity score were deleted if there were other questions with a better score that covered the intended meaning or re-written (Table 2). Simultaneously, the board members extensively revised the whole item pool for improvement. Finally, 28 items having variable scores haigh and low were deleted, 10 were re-written, and 1 was merged with another similar question. After deleting 28 items, the S-FVI/Ave items were recalculated for both clarity and importance and were found to have increased to 0.9 and 0.94, respectively, which are considered excellent FVIs. All of the retained items had an I-FVI for importance of > 0.8. Additional file 1 shows all items, I-FVI, S-FVI/Ave, and decision. The total number of retained items was 94 (Additional file 1).

**Table 2:** Items with face validity index less than 0.78 and the action taken

Items	I-FVI^a^ (Clarity)	I-FVI^a^ (Importance)	Action
1.How easy is to find appointments at a fitting time?	0.8	0.7	Delete
3.What is the nearest appointment you usually find? E.g. within a week, >1wk-3wk, >3wk- 5 wk., > 5 wk	0.5	0.8	Rewrite
4.How easy is it to reach the health facility for infertility treatment?	0.6	0.9	Delete
7.To what extent did this health facility show justice in providing services e.g. booking appointment, go into the clinic?	0.6	0.6	Delete
8.How frequent did you need an intermediary to book appointment or access health services for infertility?	0.7	0.8	Rewrite
12.What is the average waiting time you spend before entering the clinic?	0.7	0.8	Rewrite
14.To what extent did you face the problem of overcrowding while receiving health care in this facility?	0.5	0.8	Delete
20.How do you rate the cleanliness in the water closet	0.7	1.0	Delete
21.How do you rate the tidiness in this health facility in general?	0.7	1.0	Rewrite
32.Did your physician or nurse consider your preferences regarding privacy?	0.3	0.9	Delete
34.Did your physician or nurse let you know who are the health staff in the room when you receive fertility care?	0.7	0.9	Delete
36.Was there another patient with you in the same room while you were receiving fertility care?	0.7	0.9	Delete
38.Did your physician or nurse tell others about your infertility issues while you preferred not to?	0.6	1.0	Rewrite
47.How do you rate the staff communication during your treatment journey?	0.8	0.6	Delete
48.Did the healthcare team members respect each other?	0.6	0.6	Delete
49.Did you notice inappropriate behavior from any healthcare team member?	0.6	0.4	Delete
50.Did your physician or nurse tell you something during your treatment journey then you discovered it was not true?	0.6	0.9	Rewrite
51.Have you booked for an operation or procedure to be done by your physician then another one did it for you without your permission?	0.6	0.9	Delete
52.Was your physician honest about the possible outcomes of infertility care? E.g. success rate of ICSI	0.5	1.0	Delete
53.Was the healthcare team willing to tell you about errors or incidents, if happened?	0.5	1.0	Rewrite
54.To what extent can you trust your physician?	0.6	0.9	Rewrite
55.Did you notice materialistic behaviors from your physician or nurse?	0.4	0.8	Delete
56.Did you need to pay extra fees directly to your physician or nurse to receive fertility care?	0.6	1.0	Rewrite
57.To what extent did the medical team practice medicine in love and dedication?	0.6	0.8	Delete
63.Did your physician give you the prescription without examining your body?	0.7	1.0	Rewrite
70.Was your physician keen to prescribe medicines with the least side effects?	0.8	0.7	Delete
74.Have you ever felt that you extract the words from your physician by effort?	0.5	0.8	Delete
95.Did you receive infertility awareness-raising activity invitation or announcement through this health facility?	1	0.5	Delete

^a^*I-FVI* Item face validity index

### Content validity

Calculation and interpretation of the CVI for items (I-CVI) and scale (S-CVI/Ave) were conducted in the same manner as that for the FVI. Of 94 items involved in the CVI, the I-CVI ranged from 0.40–1.00, with 37 items having scores of < 0.78. The S-CVI/Ave was 0.80. The items scored < 0.78 were deleted. Additional file 1 shows the CVI and decision for each item. After deleting 37 items, the S-CVI/Ave increased to 0.90. The total number of retained items was 57. Additional file 1 shows the retained items and CVI after deletions.

### PCIQ-F draft questionnaire

The draft of our questionnaire after face and content validation contained three sections: background variables, PCIC experience variables, and a general question about the quality of infertility care in the health facility in general. Additional file 2 shows the English version of the PCIQ-F draft. This draft is ready for the remaining psychometric testing.

I. Background variables:

1. Name of the health facility being evaluated
2. Age
3. Level of education
4. Number of pregnancies
5. Number of living offspring
6. Types of treatment used
7. Duration of infertility treatment
8. Pregnancy status

II. PCIC variables, divided into 10 domains, with a total of 57 questions:

1. Accessibility (6 questions)
2. Minimizing cost (1 question)
3. Physical comfort (5 questions)
4. Privacy (5 questions)
5. Staff attitude and communication (6 questions)
6. Staff competence (3 questions)
7. Information and education (14 questions)
8. Psychological and emotional support (7 questions)
9. Continuity and coordination of care (8 questions)
10. Participation in care (2 questions)

III. General question about the quality of infertility care in the health facility in general (10-point Likert scale to maximize scale discrimination as this is the only question to assess the quality in health facility as a whole) [14].

## Discussion

This study presented the steps in developing a draft questionnaire assessing PCIC for female clients (PCIQ-F). To our knowledge, this is the first questionnaire developed in the Arabic language and from Arab and Middle Eastern women’s experiences to measure PCIC. We adopted a clear and comprehensive approach to develop the questionnaire by applying the steps proposed by Robert F. DeVellis for scale development and the recommended practices for questionnaire development and testing in the European statistical system. Our approach included two parts: 1) The first was to develop the draft questionnaire: conceptualization and operationalization, generation of an item pool, determination of the questionnaire format, initial item pool review by experts, and consideration of which validation items to include. 2) Testing and finalization of the developed questionnaire, which is beyond the scope of this paper.

Compared with the approach used for the majority of the available questionnaires measuring PCIC, our approach demonstrated the process of questionnaire development from the point of zero details. We needed to do that because we lacked a clear definition of the PCC concept from Arab and Middle Eastern client’s perspectives. A review of the available tools showed that some tools focused on other aspects of health care quality but not patient centeredness; e.g. the FertiQoL tool by Boivin et al. [8], another tool that was developed from a pre-existing general questionnaire (quality from the patient’s perspective) [12] that did not explore the concept from infertile patients’ experiences and was being used for IVF patients only. Some studies have included a questionnaire as a tool to measure the primary objective of the study (patient satisfaction and experience with IVF), so the details of questionnaire development and validation were not included [30]. The PCQ-infertility is one of the best available tools to validly and reliably measure PCIC because it was developed from qualitative study results that first defined the PCIC on the basis of the patients’ own words, then tested the psychometric properties of the developed questionnaire [11]. However that questionnaire was developed and tested on European patients only, which might not be applicable to other cultures. In addition, the PCQ-infertility is used for infertile couples together, which may overlook points that are important to one partner but not to the other, or give biased results represented by the dominant partner.

It is important to highlight some cultural and religious aspects of infertility in the Middle East where the majority of people are Arabs and Muslims. The perception of infertility as the woman’s fault is still common there and has been reported in older and recent literature despite modernization and development [31, 32]. On the other hand, although male infertility is perceived as an emasculating condition, it is usually kept as a close secret to avoid stigmatization [33]. Treatment of male infertility is mostly achieved by using assisted reproductive technologies, which are performed in the women’s body [34] and thus hides men and shows women as the cause of infertility in front of their communities, which causes more psycho-social stress in the affected women. Furthermore, the reproductive technologies that include third party donation are prohibited in Islam and therefore in most Middle Eastern countries [31]. All of these differences and others mean that adoption of a European concept and tool regarding PCIC is inappropriate and necessitates creating a concept from Arab and Middle Eastern peoples’ perspectives.

Our approach started by PCIC conceptualization mainly by IDIs, which involved the target users. Similarly, a qualitative method was preferred by the authors as an initial step to develop tools measuring patient experiences in fertility care [11, 30]. Van Empel conducted seven focus groups with 54 infertile patients to conceptualize patient centeredness [11]. We adopted IDIs rather than focus group discussion because the former is known to be more suitable for sensitive topics [30, 35, 36]. In addition, IDIs are generally thought to be preferable to other forms of qualitative and quantitative research when collecting data about patient-reported experiences [37, 38]. Some of the limitations of focus group discussion on the subject of patient experience include feeling difficulty in disclosing sensitive issues related to the experience in front of the group that could be important, giving responses that are socially acceptable rather than expressing the real opinion or experience, and the occasional challenge of giving all of the participants the same opportunity to express their experience due to the predominance of some of them [38].

Concerning the item pool, a large item pool was created to cover the concept comprehensively [14]. Large item pools were created in similar questionnaires measuring PCIC and found to be helpful [11, 36]. The literature has shown that the initial item pool should be at least twice (and preferably five times) as large as the desired final version [39, 40].

Regarding the format of the PCIQ-F, we followed several tips to achieve higher measurement quality and eliminate confusion. These tips included dominating closed-ended questions, item-specific rating rather than using the simple Likert rating scale, including both positive and negative items without using confusing negative phrasing, and avoiding filtering questions because they are confusing [14, 23, 24].

A literature review has shown that scale characteristics have an impact on data quality; e.g., rating scales provided a lower number of item-nonresponses and higher reliability than those of continuous scales, and item-specific scales provided higher measurement quality than binary agree–disagree scales [23].

Since we started with a very large item pool, it was necessary to reduce it and decide which items should be included in the draft questionnaire, which is the content validation. Many methods have been used by researchers to conduct content validation. The CVI is widely used because it gives a score for each item to enable developers to make decisions in addition to its simplicity in calculation, reporting, and understanding [29, 41].

However, it can be argued that there is a possibility of chance agreement when using the CVI, so additional measures, such as the inter-rater agreement has to be undertaken. Pilot et al. concluded that a CVI score of ≥0.78 generally indicates a low possibility of chance agreement, especially if the number of experts is ≥10 [41].

The limitation of our approach was that it included participants from a single city rather than from an entire country or multiple countries. We tried to overcome this by choosing a central city that provides tertiary health care for a wide area in the kingdom. The women with infertility included in this study were treated in Jeddah, but some of them lived outside Jeddah. In addition, we selected this group from different Arab countries in the Middle East. The diversity of our sample concerning nationality, residency, infertility features, and hospitals visited made participant selection a bit difficult and time consuming, but our aim was to make it more representative. After psychometric testing, the final questionnaire could be tested on other Arab country populations to ensure its validity and suitability for that group.

## Conclusions

The outcome of this study was the PCIQ-F draft questionnaire, which is ready for the questionnaire testing step. We expect that some items will be dropped after formal validation based on the results of psychometric testing. However, reporting these steps in the questionnaire development is of paramount importance because it shows other researchers a clear and reproducible method for development of new tools of this type.

## Supplementary Information

**Additional file 1:** Item pool with face validity index for calrity and importance, and the decision taken. FVI and S-FVI/Ave after deleting items. Content validity index with items decision. The items for draft patient-centered infertility care questionnaire for female clients (PCIQ-F), with item and domain content validity index.

**Additional file 2:** Patient-Centered Infertility Care for Female Clients (PCIQ-F) Draft Questionnaire.

## Data Availability

All data generated or analyzed during this study are included in this published article and its supplementary information files.

## Abbreviations

CVI: Content validity index
FVI: Face-validity index
IDI: In-depth interviews
IVF: In vitro fertilization
PCC: Patient-centered care
PCIC: Patient-centered infertility care
PCIQ-F: Patient-centered infertility questionnaire for female clients
PCQ-infertility: Patient centeredness questionnaire-infertility
