# Supplementary material for: Patient-centered infertility questionnaire for female clients (PCIQ-F): part I: questionnaire development
Source: BMC Med Res Methodol. 2021 Sep 20;21:188. doi: 10.1186/s12874-021-01376-w (PMC8454158; doi:10.1186/s12874-021-01376-w)
Supplement: Supplementary file 2 — Additional file 2: Patient-Centered Infertility Care for Female Clients (PCIQ-F) Draft Questionnaire. [file 12874_2021_1376_MOESM2_ESM.doc]

**Patient-Centered Infertility Care for Female Clients (PCIQ-F) Draft Questionnaire**

Dear experts,

This is a draft version of Patient-centered infertility care questionnaire for female clients (PCIQ-F). This questionnaire targets Arab infertile women who sought infertility care in Jeddah, KSA within the last 6 months prior to the survey time. The questionnaire composed of 3 sections; personal data, patient centered infertility care, and overall evaluation of infertility care quality. All questions are multiple choice ones apart from few questions in Section I which include writing short answers (numbers) about yourself. The total number of items is 66. Nothing is right or wrong, the questions are asking about your experience with infertility care. Your confidentiality will be insured and none of the identifying information is added. The result of this survey will help to validate this questionnaire and produce its final version. We are welcoming your constructive comments on each item and at the end of this draft version.

We much appreciate your time and effort

| **Section I: Personal data** |
| --- |
| 1. The name of health facility being evaluated ……………………………………….. |
| 1. Age by years …………………………………………………………. |
| 1. level of education: Primary school 🞎 Secondary school 🞎 University or higher🞎 |
| 1. Number of pregnancies ……………………………… |
| 1. Number of living offspring …………………………….. |
| 1. types of treatment used: Ovulation induction 🞎 Intrauterine insemination 🞎 In vitro fertilization 🞎 Surgery 🞎 Others………………… |
| 1. duration of infertility treatment ………………………………………………………………… |
| 1. pregnancy status: Pregnant 🞎 Not pregnant 🞎 |

| **Section II: Patient-centered infertility care questions** | |
| --- | --- |
|  | **Accessibility** |
|  | Have you given appointment at the time that is out of the purpose of your visit (like for ovulation monitoring)? |
|  | How smooth is it to book an appropriate appointment? |
|  | How smooth was the process to receive health services; e.g. open a file, medications dispension, referral to do image or lab tests …? |
|  | Did you receive a contact number to use in case you need urgent help?   1. Yes 2. No |
|  | Have you needed to communicate with your physicians through phone like by calls or WhatsApp and couldn’t not reach them? |
|  | How frequent did you go for infertility care with a prescheduled appointment and did not receive the service on time? |
|  | **Minimizing cost** |
|  | How do you rate the cost of fertility care in this health facility? |
|  | **Physical comfort** |
|  | How do you rate the cleanliness in this health facility in general? |
|  | How tidy is this health facility in general? |
|  | How do you rate the comfort in this health facility in general? |
|  | Did you need assistance while in this health facility but you did not receive help? |
|  | How do you rate the nursing care you received? |
|  | **Privacy** |
|  | Did this health facility provide a female physician to examine you if you asked for that? |
|  | To what extent did you feel that your privacy was respected? |
|  | Did your physician or nurse take your permission before allowing other persons to go into the clinic or procedure room like trainees, physicians…etc? |
|  | Did your physician or nurse talk about your infertility issues to others while you preferred not to? |
|  | How frequent did your physician involve your husband in fertility care; his presence, explanation of your issues,…? |
|  | **Staff attitude and communication** |
|  | To what extent did the healthcare team deal with you with respect and dignity? |
|  | Have you ever changed your physician due to his/her poor communication or performance? |
|  | Did your physician spend adequate time with you? |
|  | How do you rate the physicians' communication during your treatment journey? |
|  | Did your physician or nurse tell you something then you discovered it was not true? |
|  | Did your physician or nurse asked you to pay extra fees directly to them for fertility care? |
|  | Did your physicians ask about history of your illness adequately? |
|  | Did your physician give you curative solutions for your infertility problem? |
|  | Did your physician consider all the aspects related to your infertility like psychological, life style factor…? |
|  | **Information and education** |
|  | To what extent did your physician help you to understand your infertility problem fully? |
|  | Did you need to search or ask other persons (other than your physician) to understand your infertility problem and its treatment more? |
|  | Did your physicians tell you about all treatment options suitable for you? |
|  | Did you receive adequate information about each of the available treatment options? |
|  | Did your physician explain for you what do expect before, during and after any procedures? |
|  | Did you receive clear home care instructions, e.g. how to use the medicines? |
|  | Did your physician discuss with you clearly the follow up plan; what will be in the next visit? |
|  | Were you informed about common medicines side effects before starting using them? |
|  | How do you rate the information you received during fertility treatment journey in general? |
|  | Did your physician discuss what you should do or avoid during your treatment period? |
|  | Did your physician explain for you adequately what are the possible complications of your complaint especially if not treated? |
|  | To what extent did you understand the information given by healthcare team? |
|  | Did your physician and nurse answer your queries comprehensively? |
|  | Did your physician provide awareness-raising concerning fertility issues for you and your husband? |
|  | **Psychological and emotional support** |
|  | Did your physician listen to you carefully? |
|  | Did your physician allow you to speak without interrupting you early? |
|  | Did you feel that your physician considered your personal and special situation when providing care? |
|  | To what extent the medical team show empathy when dealing with you? |
|  | Did your physician give you a depressive talk regarding your fertility issues? E.g. “It is impossible to get pregnant” |
|  | Did you have adequate psychosocial support during treatment journey? |
|  | To what extent did your physician or nurse motivate you? |
|  | **Continuity and coordination of care** |
|  | Did you feel that your physician had checked your medical record before starting with you? |
|  | How often did you need to repeat the same story to your physicians during infertility treatment journey? |
|  | To what extent did your physician consider your husband fertility issues? |
|  | Did your physician share with you a clear treatment plan from the start? |
|  | Did your physician provide ongoing follow up of your case? |
|  | Did you have one physician responsible of decision-making and follow up of your case? |
|  | Did your physicians involve other specialist in your case e.g. dietician, psychologist, …? |
|  | Did you pause unnecessarily between a step to the next during fertility care? |
|  | **Participation in care** |
|  | Did your physician share you updates in your treatment plan? |
|  | Did your physician encourage you to participate in the decisions about your treatment? |
| **Section III: Overall evaluation of infertility care quality** | |
|  | How do you rate the quality of infertility care in this health facility in general? 1 is very poor and 10 is excellent. |

**Thank you for your time**
